# Comparing Proteolytic Fingerprints of Antigen-Presenting Cells during Allergen Processing

**DOI:** 10.3390/ijms18061225

**Published:** 2017-06-08

**Authors:** Heidi Hofer, Tamara Weidinger, Peter Briza, Claudia Asam, Martin Wolf, Teresa E. Twaroch, Frank Stolz, Angela Neubauer, Elfriede Dall, Peter Hammerl, Alain Jacquet, Michael Wallner

**Affiliations:** 1 Department of Molecular Biology, University of Salzburg, Salzburg 5020, Austria; heidi.hofer@sbg.ac.at (H.H.); tamara.weidinger@stud.sbg.ac.at (T.W.); peter.briza@sbg.ac.at (P.B.); claudia.asam@sbg.ac.at (C.A.); martin.wolf@sbg.ac.at (M.W.); elfriede.dall@sbg.ac.at (E.D.); peter.hammerl@sbg.ac.at (P.H.); 2 Biomay AG, Vienna 1090, Austria; teresa.twaroch@gmx.at (T.E.T.); f.stolz@biomay.com (F.S.); a.neubauer@biomay.com (A.N.); 3 Department of Medicine, Faculty of Medicine, Chulalongkorn University, Bangkok 10330, Thailand; alain.j@chula.ac.th

**Keywords:** allergen proteolysis, Bet v 1, Amb a 1, Der p 1, Der p 2, proteases from dendritic cells, proteases from macrophages, proteases from B cells, degradome assay

## Abstract

Endolysosomal processing has a critical influence on immunogenicity as well as immune polarization of protein antigens. In industrialized countries, allergies affect around 25% of the population. For the rational design of protein-based allergy therapeutics for immunotherapy, a good knowledge of T cell-reactive regions on allergens is required. Thus, we sought to analyze endolysosomal degradation patterns of inhalant allergens. Four major allergens from ragweed, birch, as well as house dust mites were produced as recombinant proteins. Endolysosomal proteases were purified by differential centrifugation from dendritic cells, macrophages, and B cells, and combined with allergens for proteolytic processing. Thereafter, endolysosomal proteolysis was monitored by protein gel electrophoresis and mass spectrometry. We found that the overall proteolytic activity of specific endolysosomal fractions differed substantially, whereas the degradation patterns of the four model allergens obtained with the different proteases were extremely similar. Moreover, previously identified T cell epitopes were assigned to endolysosomal peptides and indeed showed a good overlap with known T cell epitopes for all four candidate allergens. Thus, we propose that the degradome assay can be used as a predictor to determine antigenic peptides as potential T cell epitopes, which will help in the rational design of protein-based allergy vaccine candidates.

## 1. Introduction

Over a decade ago, Delamarre et al. suggested that differential processing of antigens in lysosomal compartments may directly influence the quality of the subsequent immune response [1]. In general, antigenic peptides can be presented either on major histocompatibility complex (MHC) class I or class II molecules to CD8^+^ or CD4^+^ T cells, respectively. MHCI molecules are found on almost all cell types and present peptides derived from intracellular antigens, whereas MHCII complexes are constitutively expressed only on professional antigen-presenting cells (APCs) and primarily display extracellular antigens [2]. Professional APCs include dendritic cells (DCs), B cells, macrophages, and thymic epithelial cells, the latter playing a central role in controlling T cell development and T cell tolerance [3,4]. MHCII molecules are assembled with a chaperone protein called the invariant chain (Ii) in the endoplasmic reticulum, which is trimmed and cleaved in the endosome where the residual part of the invariant chain (CLIP) is ultimately replaced by antigenic peptides—a process regulated by HLA-DM (human leukocyte antigen DM) [2]. For antigen uptake, APCs use different mechanisms such as macropinocytosis, receptor-mediated endocytosis, or phagocytosis to internalize extracellular material into vesicles where antigen processing is initiated. Of note, 20–30% of antigenic peptides eluted from MHCII molecules have an intracellular origin and are transferred to endolysosomal compartments via mechanisms of autophagy [3]. Following internalization, antigens are proteolytically processed in endolysosomal compartments and thereafter loaded on the MHCII complex and presented to T cells, which eventually initiate T cell activation. This process is fundamental for the development of an adaptive immune response.

To date, 25% of the population in industrialized countries are affected by allergies, whereas allergen immunotherapy based on formulated allergen extracts represents the only treatment option, which may eventually modify the cause of the disease. However, the use of allergen extracts for therapeutic interventions is a matter of controversy, which led to the suggestion of replacing extracts with purified allergens or allergen epitopes [5]. Yet, the design of allergy vaccines requires a good knowledge of B as well as T cell-reactive regions on the allergenic molecules. Antibody epitope mapping strategies are applied to identify B cell epitopes, whereas the identification of T cell epitopes is primarily achieved by proliferation assays using short overlapping peptides covering the entire sequence of the allergen [5,6]. In addition, an in vitro assay has been developed that mimics the natural proteolytic processing of allergens in the endolysosomal compartments of APCs. For the assay, a cocktail of proteases isolated from a murine DC line is combined with allergenic proteins and protein degradation is monitored by mass spectrometry. The authors suggested that this protease mix could be applied as a surrogate for human DC-derived proteases to identify T cell-reactive regions on allergens [7]. In fact, a study with the major birch pollen allergen Bet v 1 demonstrated that the in vitro generated Bet v 1-derived peptides showed a precise overlap with naturally processed T cell-reactive peptides isolated from DCs as well as peptides obtained from T cell epitope mapping of Bet v 1 [8,9].

We therefore sought to compare the degradation pattern of proteases isolated from different APCs, i.e., macrophages, B cells, and DCs. Four different major inhalant allergens from ragweed (Amb a 1) and birch pollen (Bet v 1), as well as house dust mites (Der p 1 and 2) were selected and produced as recombinant proteins. Thereafter, the allergens were combined with endolysosomal proteases, whereas proteolysis was monitored by protein gel electrophoresis and mass spectrometry. Moreover, we tested whether the activation of APCs with bacterial endotoxin had an influence on the proteolytic pattern or speed.

## 2. Results

### 2.1. Expression and Purification of Recombinant Allergens

**Figure 1:**
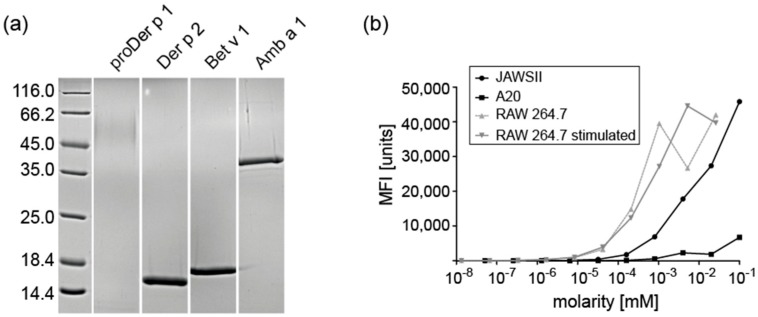
(**a**) 15% SDS-PAGE of purified allergens; 1 μg recombinant proteins were loaded per lane; (**b**) The protease activity of endolysosomal fractions was determined using the fluorescent substrate Z-Phe-Arg-AMC.

The major inhalant allergens Bet v 1 from birch, Amb a 1 from ragweed, as well as proDer p 1 and Der p 2 from house dust mites were produced either in *E. coli* (Bet v 1 and Der p 2) or *P. pastoris* (Amb a 1 and proDer p 1). As Der p 2 has three internal disulphide bonds, an *E. coli* strain capable of forming cysteine bonds in the bacterial cytoplasm was selected. The correct formation of cysteine bonds was verified by mass spectrometry (data not shown). On SDS-PAGE, the band of proDer p 1 appeared blurry as the protein is hypermannosylated and therefore not migrating as a homogenous specimen. All recombinant allergens were purified to homogeneity (Figure 1a) and characterized physicochemically prior to degradation analyses (data not shown).

### 2.2. Subcellular Fractionation of Endolysosomes and Characterization of Proteolytic Activity

Endolysosomes were isolated by differential centrifugation from three different APC cell lines, i.e., A20 B cells, JAWSII DCs, and RAW 264.7 macrophages, respectively. Total protein content was determined by Bradford assays. From 2 × 10^7^ cells, we obtained 630 μg of JAWSII, 165 μg of RAW 264.7, and 99 μg of A20 endolysosomal proteins. To investigate whether the activation status of APCs could influence the proteolytic activity, we stimulated RAW 264.7 cells with bacterial endotoxin (LPS) for 24 h. Thereafter, cells were harvested and counted. Indeed, the total endolysosomal protein content of RAW 264.7 increased to 270 μg/2 × 10^7^ cells after activation.

To determine the relative proteolytic activity of endolysosomal proteases, we used the substrate Z-Phe-Arg-AMC, which can be cleaved by cysteine proteases, i.e., cathepsin B, F, K, L, and S, to a fluorescent product [10,11]. RAW 264.7 macrophages displayed the highest proteolytic activity using this substrate, followed by JAWSII- and A20-derived endolysosomal proteases. The relative proteolytic activity of RAW 264.7 proteases was 6.3 times higher than the activity of endolysosomal proteases from JAWSII cells and 956 times higher than the activity of A20, whereas the difference between JAWSII and A20 was a factor of 151 (Figure 1b). However, there was no difference in proteolytic activity between resting and activated RAW 264.7 cells (0.059 vs. 0.056 mM).

### 2.3. Endolysosomal Degradations of Inhalant Allergens

**Figure 2:**
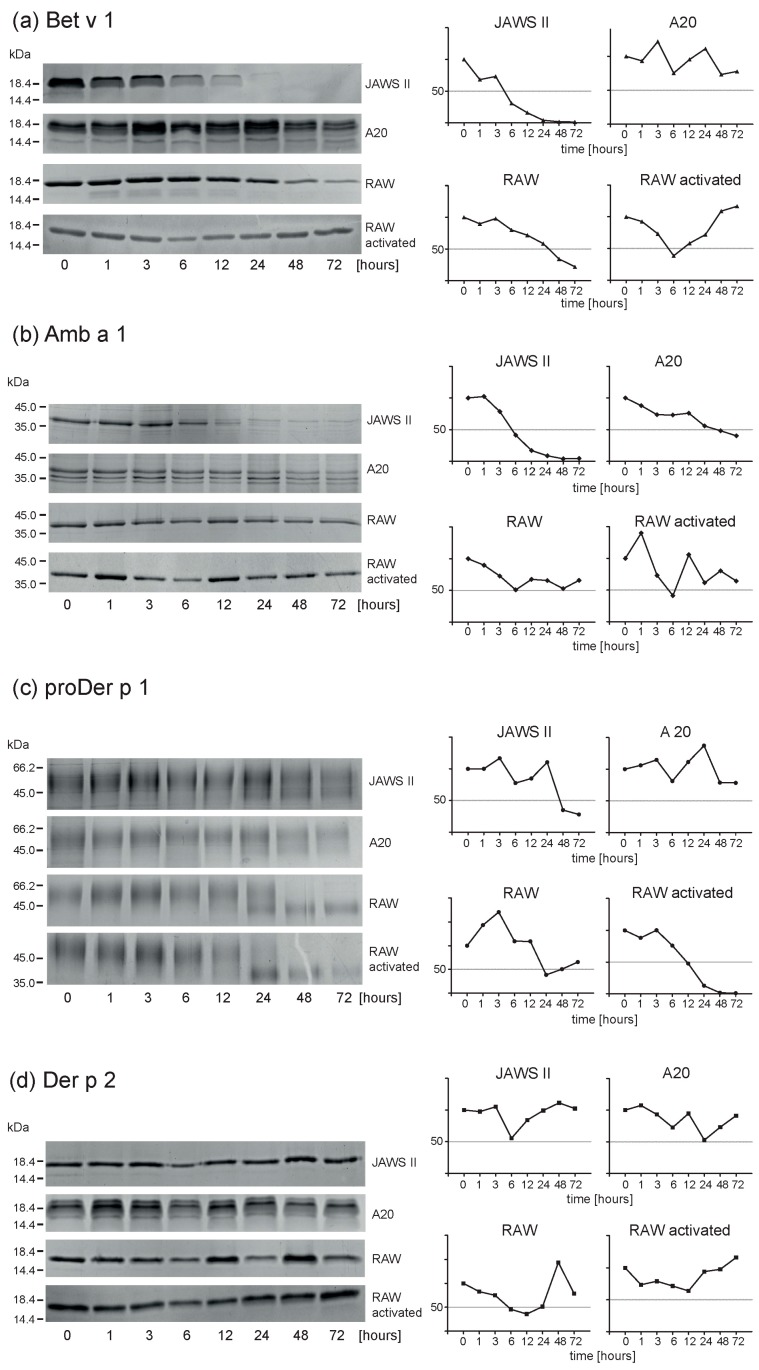
Fifteen percent SDS-PAGE analyses of proteolytically processed allergens. The bands representing intact protein were scanned using the Bio-Rad Chemidoc™ MP Imaging System and evaluated with the Bio-Rad Image Lab Software tool. (**a**) Bet v 1, (**b**) Amb a 1, (**c**) proDer p 1, and (**d**) Der p 2 were digested with endolysosomal proteases from different APCs.

For proteolytic processing of inhalant allergens, 7.5 arbitrary units of JAWSII- as well as RAW 264.7-derived endolysosomal proteases were applied for each degradation, whereas we used 0.01 arbitrary units of A20 proteases. This was necessary, as the endolysosomal A20 proteins showed a very low proteolytic activity/μg and as the amount of proteins could not have been increased without increasing the volume of the degradome samples substantially. Degradations were performed in a time-dependent manner under reducing conditions at 37 °C and a pH of 4.8. Reactions were stopped by heat inactivation of the protease/allergen mixture [7]. The low pH is intended to resemble the pH value of late endosomes [12]. Subsequently, 10 μL aliquots of the degraded samples were analyzed by SDS-PAGE and the decrease of intact protein was measured densitometrically (Figure 2). Bet v 1 was very efficiently degraded by JAWSII proteases. After 6 h, 70% of the intact protein had already been processed, whereas for RAW 264.7 after 6 h, 80% of Bet v 1 remained still intact and for A20 we could hardly determine any degradation of the protein. Amb a 1 was efficiently degraded by JAWSII (58% degradation after 6 h), and to a lesser extent by RAW 264.7 (48% degradation after 6 h) and A20 (27% degradation after 6 h). The recombinant dust mite allergen proDer p 1, a hypermannosylated glycoprotein stabilized by three intermolecular disulphide bridges, [13], was found to be very resistant to endolysosomal proteolysis (>80% intact protein after 24 h for JAWSII and A20). Surprisingly, digestions with RAW 264.7 proteases resulted in approximately 60% degradation of proDer p 1 after 24 h. Though the dust mite allergen Der p 2 is not glycosylated, this 14 kDa protein has also three intramolecular disulphide bridges stabilizing the predominantly beta-sheet structure of the allergen [14]. Thus, in our experimental setup we could hardly measure any decrease in intact Der p 2 over time with any of the three endolysosomal fractions used.

### 2.4. Activated Macrophages Display Identical Proteolysis Pattern as Resting Cells

Compared to resting cells, activated macrophages showed a very similar activity pattern (Figure 2). Endolysosomal enzymes from activated as well as resting macrophages degraded Bet v 1 and Amb a 1 rather slowly (both allergens showed approx. 40% degradation for either cell line after 24 h), whereas proDer p 1 was degraded efficiently (approx. 60% degradation for either activated or resting macrophages after 24 h). Again, Der p 2 showed hardly any reduction of intact protein.

### 2.5. Mass Spectrometric Analyses of Degradation Patterns Revealed No Differences Between Cell Lines

**Figure 3:**
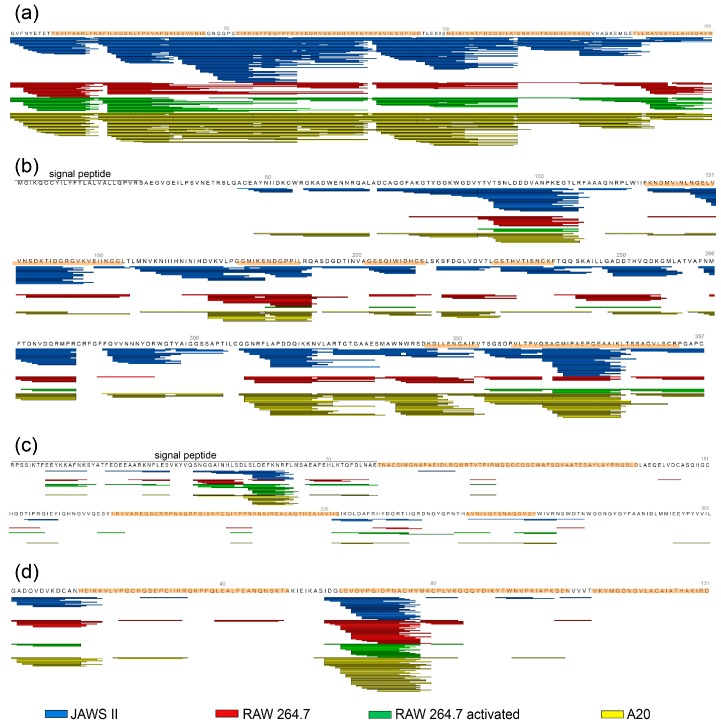
Endolysosomal degradations of purified allergens were analyzed by mass spectrometry (MS) after 6 h. Peptides identified by MS are represented as bars. Protein sequence areas, which contain published T cell epitopes, are highlighted in orange. The following protein sequences were used: Bet v 1.0101 (**a**), Amb a 1.0301 (**b**), proDer p 1.0102 (**c**), and Der p 2.0103 (**d**).

We compared the degradation patterns of all four inhalant allergens processed with endolysosomal enzymes from different cell lines, as well as proteases from activated and resting macrophages at two time points (6 and 24 h) (Figure 3 and Figure S1). For Bet v 1, we found that after 6 h most peptides were produced by JAWSII proteases. Though we did not observe a substantial decrease in intact Bet v 1, we also obtained almost the same amount of different peptides with the A20 protease fraction as with JAWSII cells. The peptide patterns of activated and resting RAW 264.7 appeared to be identical. After 24 h of proteolysis, the patterns between the cell lines did not change, although we found differences in the number of peptides detected for each cell line.

For Amb a 1 the picture was similar and we obtained conserved peptide stacks for each time point. After 6 h most peptides were acquired from JAWSII degradations, whereas after 24 h the A20 proteases produced a large amount of antigenic peptides. Again, we could not find any difference between activated and resting macrophages. Despite the fact that macrophage-derived proteases were more efficient in degrading proDer p 1, we found very similar peptide patterns after 6 and 24 h for all cell lines and activation states. For Der p 2 we hardly observed any degradation of full-length protein on SDS-PAGE. The degradation patterns were virtually identical, independent of the protease source. In general, proteolytic digestions of proDer p 1 and Der p 2 resulted in less potentially T cell-reactive peptides compared to proteolysis of Bet v 1 or Amb a 1; however, we also found peptide stacks for these proteolytically stable allergens. Natural Der p 1 is synthesized in mites as an inactive pre-pro enzyme (pre-proDer p 1), which has to undergo maturation leading to full proteolytically active Der p 1 [15]. As we used proDer p 1 for our degradome experiments, we found that the propeptide was, in general, less stable than the covalently linked mature allergen; thus we obtained more peptides from this part of the recombinant protein. Nevertheless, we also measured peptides derived from the sequence of mature Der p 1. Of note, at an acidic pH (pH 4), the unfolding of the propeptide is initiated followed by a multi-step auto-degradation catalyzed by Der p 1 itself, which eventually leads to the maturation of the allergen under reducing conditions [16]. Recently, it was demonstrated that at pH 4.8 the autocatalytic processing of unglycosylated proDer p 1 (N16pQ/N52Q) reached only 20% of the maturation observed at pH 4. Moreover, the full-length wild-type recombinant propeptide produced in *E. coli* (unglycosylated, equivalent to N16pQ) could not be hydrolyzed by Der p 1 at pH 4.8 but only at pH 4 [17]. In our study, we evaluated the endolysosomal degradation of glycosylated wild type proDer p 1. We cannot fully exclude that residual auto-maturation of Der p 1 occurs under our experimental conditions. However, the presence of glycosylations and particularly glycan residues present in the propeptide structure negatively influences the maturation process of proDer p 1 [18]. Thus, only a very limited amount of active Der p 1 can be generated, which could also degrade endolysosomal proteases. However, we do not have evidence that this could explain the low levels of Der p 1 degradation seen in our assays.

## 3. Discussion

APCs internalize and process extracellular antigens, and antigenic peptides are subsequently presented on MHCII, eventually triggering T cell activation. Therefore, a variety of proteases is present in endolysosomal compartments of APCs. The process of endolysosomal degradation is generally considered as rather unspecific and is substantially determined by the accessibility of cleavage points on the antigen available to the active sites of proteases. The low pH as well as the reduction of disulphide bonds promote antigen proteolysis [19]. For many antigens, the role of the various endolysosomal proteases in this context is probably redundant, although some examples (i.e., tetanus toxin) show that a specific enzyme (in that case asparaginyl endopeptidase) is required to initiate the processing of an antigen [20]. However, there are also examples where the lack of a specific protease can influence antigen processing. In the case of the myelin basic protein, the antigen harbors a cleavage site for asparaginyl endopeptidase (AEP) in a dominant T cell epitope, thus the presentation of this specific epitope inversely correlates with the expression of the protease [21].

The majority of endolysosomal proteases belong to the aspartyl and cysteine protease families of endo- and/or exopeptidases [7]. In fact, the expression patterns of endolysosomal proteases seem variable and cell type-specific, as it has been reported that APCs differ significantly in their ability to degrade antigens. For instance, macrophages express substantially higher amounts of endolysosomal proteases compared to B cells or DCs [1]. This could also imply that cell line-derived APCs could have different protease expression patterns than in vivo isolated cells. However, in a study published in 2011, Egger et al. demonstrated that JAWSII cells contained all important proteases necessary for antigen processing [7]. Therefore, they compared the proteomes of microsomal fractions isolated from the murine DC cell line JAWSII with murine bone marrow- as well as human blood-derived DCs by mass spectrometry. Moreover, they performed degradome assays of multiple different antigens using proteases from these three sources and found no differences in the proteolytic activity. In the end, the authors managed to develop a straightforward and scalable assay mimicking the complex events of proteolysis within endolysosomal compartments based on cell line-derived microsomes. Based on these findings, we sought to compare the proteolytic pattern of different APCs (macrophages, DCs, and B cells) from well-established cell lines using a panel of four major inhalant allergens from pollen as well as dust mites. First, we isolated endolysosomal fractions of different APCs and compared the proteolytic activity using the substrate Z-Phe-Arg-AMC, which is specific for cysteine cathepsins [22]. As mentioned, cysteine proteases depict a major proportion of all endolysosomal proteases, thus the cysteine protease activity was used as a surrogate to determine the activity of endolysosomal proteases. We found that macrophages showed the highest proteolytic activity, followed by DCs and B cells with by far the lowest activity in this assay. We also compared the proteolytic activity of activated vs. resting macrophages and, when normalized to the total protein concentration, we could not measure any difference in the proteolytic activity. However, the total amount of endolysosomal proteins of activated macrophages was almost twice as high as that from resting cells. Thereafter, we performed degradation studies to compare the proteolysis pattern of DCs, B cells, as well as activated and resting macrophages. The degradation patterns were analyzed by mass spectrometry and identified peptides were assigned to sequence areas of the respective allergen. Despite minor differences in the processing of the four model allergens, the overall degradation patterns were extremely similar. Moreover, there was no difference between resting and activated cells in terms of proteolysis pattern. This indicates that the protein structure rather than the source of endolysosomal proteases determined the fate of the allergens in our study. The finding also supports the idea that antigen processing can be studied with endolysosomal proteases isolated from a single cell line.

As proteolytically processed antigens represent the source material for T cell-reactive peptides, we further investigated whether the allergen peptides would match previously identified T cell epitopes (Figure 3) [23,24,25,26,27,28,29,30,31,32]. Despite the fact that the pollen allergens Bet v 1 and Amb a 1 are processed rapidly, whereas the mite allergens Der p 1 and Der p 2 are degraded slowly, we found a very good overlap of the proteolytically processed peptides with known T cell epitopes for all four candidate allergens.

As natural allergens often represent heterogeneous mixtures of multiple isoforms [33,34,35,36], which cannot easily be separated by chromatographic procedures, we restricted ourselves to the use of well-characterized recombinant allergens. Therefore, the assignment of processed peptides to a unique protein sequence becomes feasible, which allowed the exact determination of degradation patterns. We propose that the degradome assay can be used as a predictor to determine antigenic peptides as potential T cell epitopes. Moreover, the method is independent of the APC cell line used for harvesting the endolysosomal proteases. Therefore, the method could not only be a valuable tool to determine the immunogenicity of allergens but also to predict T cell-reactive epitopes on allergenic proteins [1,7,9]. This shall help in the rational design of candidate molecules for allergen immunotherapy in the future.

## 4. Materials and Methods

### 4.1. Production of Recombinant Allergens

The birch pollen allergen Bet v 1.0101, termed Bet v 1 throughout the manuscript, was produced as recombinant protein in *E. coli* and purified to homogeneity [9].

The ragweed allergen Amb a 1.0305, termed Amb a 1 above, was supplied by Biomay AG, Vienna, Austria. The protein was expressed in *P. pastoris* and purified from culture supernatants using a two-step chromatographic procedure.

A stable pro form of the allergenic cysteine protease Der p 1 from the house dust mite species *Dermatophagoides pteronyssinus* was produced in *P. pastoris* as previously described [13]. In brief, proDer p 1.0102, termed proDer p 1 throughout the manuscript, was expressed and secreted into the culture medium. The purification from the supernatant was performed by anion-exchange chromatography at pH 9.0. Fractions containing proDer p 1 were pooled, concentrated, and a polishing step was performed by gel filtration chromatography.

The dust mite allergen Der p 2.0103, termed Der p 2 throughout the manuscript, was produced in *E. coli*. Therefore, a synthetic gene of Der p 2, obtained by ATG Biosynthetics, Merzhausen, Germany, was cloned into the pET28b vector system. Subsequently, bacteria were transformed and the protein was expressed at 37 °C. After harvesting the cells, Der p 2 was extracted from the lysate in 50 mM Tris pH 8.5 and 0.5 M urea. Following acidic precipitation at pH 4.0, the allergen-containing supernatant was loaded onto an SP-FF column (GE Healthcare, Little Chalfont, UK) and purified with a gradient to 50 mM Tris pH 4.0, 0.5 M urea, and 0.5 M NaCl.

All proteins were either lyophilized or frozen in appropriate buffers and stored at −20 °C.

### 4.2. Cell Culture

The B cell line A20 (ATCC^®^ TIB-208™) was cultured in RPMI-1640, and supplemented with 10% FCSi, 50,000 U penicillin, 50 mg streptomycin, 2 mM l-glutamine, 25 mM HEPES, 2.5 mM Na-pyruvate (all PAN-Biotech GmbH, Aidenbach, Germany), and 50 μM β-mercaptoethanol. Sub-culturing was performed at 5% CO_2_ and 37 °C according to ATCC guidelines [37]. In brief, cells were harvested by centrifugation at 250 g for 5 min; after the aspiration of the supernatant, the cells were expanded in double the volume of fresh medium.

The macrophage cell line RAW 264.7 (ATCC^®^ TIB-71™) [38] was cultured in DMEM (Dulbecco´s Modified Eagle Medium) high glucose, and supplemented with 5% FCSi, 50,000 U penicillin, 50 mg streptomycin, 2 mM l-glutamine, 10 mM HEPES, 1 mM Na-pyruvate (all PAN-Biotech GmbH), and 50 μM β-mercaptoethanol. After carefully removing the adherent cells from the bottom of the flask, all cells were harvested by centrifugation at 250 g for 5 min. RAW 264.7 cells were sub-cultured at a ratio of 1:5 once per week. The activation of RAW 264.7 cells was performed by the addition of 100 ng/mL lipopolysaccharide (Sigma, St. Louis, MO, USA) to the culture medium for 24 h.

The dendritic cell line JAWSII (ATCC^®^ CRL11908™) was cultured in MEM (Minimum Essential Medium) α-modification with l-glutamine, and supplemented with 15% FCSi, 1 mM Na-pyruvate (all PAN-Biotech GmbH), and 5% GM-CSF supernatant [7]. Cell cultures were performed according to ATCC instructions and as previously described [39].

### 4.3. Endolysosomal Fractionation

The endolysosomal protein fractions of different cell lines were obtained by differential centrifugation as previously described [1,7]. In brief, 1 × 10^8^ A20, or 2 × 10^7^ RAW 264.7 and JAWSII cells, respectively, were resuspended after two washing steps in 5 mL with 250 mM sucrose, 10 mM Tris-acetate, with a pH of 7.0, disrupted using a Dounce glass tissue grinder, and centrifuged for 10 min at 6000× *g*. Thereafter, the supernatant was centrifuged for 1 h at 100,000× *g* and the resulting pellet was resuspended in 150 μL with 250 mM sucrose, 10 mM Tris-acetate, with a pH of 7.0. Endolysosomal proteases were released by five repeated freeze/thaw cycles in liquid nitrogen and at 37 °C, respectively, and stored at −70 °C. The total protein concentration of endolysosomal fractions was determined by Bradford assay [40].

### 4.4. Determination of Proteolytic Activity

The protease activity of the endolysosomal fraction of each cell line was determined using the fluorescent substrate Z-Phe-Arg-AMC (Bachem Holding GmbH, Budendorf, Switzerland), which can be cleaved by cysteine proteases, i.e., cathepsin B, F, K, L, and S [10,11]. Therefore, endolysosomal proteases were serially diluted in 10 mM citrate buffer with a pH of 4.8, 0.2 mM dithiothreitol (DTT), supplemented with 50 μM Z-Phe-Arg-AMC, and relative fluorescent units were determined at an excitation wavelength of 380 nm and an emission wavelength of 460 nm using a Tecan Infinite M200 plate reader (Tecan Austria GmbH, Grödig, Austria). Results were plotted as RFUs (relative fluorescent units) at a given concentration. The relative proteolytic activity of endolysosomal fractions was calculated as the protein concentration required to obtain 5000 RFUs.

### 4.5. Degradation Assays

After assessing the proteolytic activity of the endolysosomal fractions of the different cell lines, degradation assays were performed as described [7]. Briefly, 5 μg protein were mixed with 7.5 μg JAWS II, 15 μg A20, or 1.2 μg RAW 264.7 endolysosomal proteases, respectively, in a volume of 20 μL with 10 mM citrate buffer with a pH of 4.8, 0.2 mM dithiothreitol. Reactions were incubated at 37 °C and stopped after 0, 1, 3, 6, 12, 24, 48, and 72 h, by denaturation at 95 °C for 5 min. Samples were stored at −20 °C and degradation profiles were analyzed by SDS-PAGE. After staining with Coomassie Brilliant Blue R-250, SDS-gels were evaluated using the Bio-Rad Chemidoc™ MP Imaging System and the Bio-Rad Image Lab Software (Bio-Rad, Hercules, CA, USA).

### 4.6. Mass Spectrometry

Samples digested with endolysosomal proteases were desalted using C18 ZipTips (EMD Millipore, Billerica, MA, USA). Resulting peptides were separated by reverse-phase nano-HPLC (Dionex Ultimate 3000, Thermo Fisher Scientific, Bremen, Germany, column: PepSwift Monolithic Nano Column, 100 μm × 25 cm, Dionex), where the column was eluted with an acetonitrile gradient (Solvent A: 0.1% (*v/v*) FA/0.01% (*v/v*) TFA/5% (*v/v*) ACN; solvent B: 0.1% (*v/v*) FA/0.01% (*v/v*) TFA/90% (*v/v*) ACN; 5–45% B in 60 min) at a flow rate of 1 μL/min at 55 °C. The peptides were analyzed by a Q Exactive Orbitrap mass spectrometer (Thermo Fisher Scientific) directly coupled to the HPLC. Capillary voltage at the nano-electrospray head was 2 kV. The instrument was tuned for maximum sensitivity. For peptide assignments, a top 12 method was used with the normalized fragmentation energy at 27%. Survey and fragment spectra were analyzed with Proteome Discoverer version 1.4 with SequestHT as search engine (Thermo Fisher Scientific) or PEAKS Studio 8 (Bioinformatics Solutions, Waterloo, ON, Canada), respectively. Searches were conducted with single allergen sequences. Only peptides with high confidence scores (XCorr ≥ 2.3 for SequestHT, −10lgP ≥ 35 for PEAKS) were considered.

## 5. Conclusions

In the present study, endolysosomal processing of major inhalant allergens was studied in vitro. Therefore, proteases were isolated from DCs, macrophages, and B cells, respectively. Whereas the overall proteolytic activity of specific endolysosomal fractions differed substantially, the degradation patterns of the digested allergens corresponded well. Thus, the protein structure rather than the source of proteases seemed decisive for antigen processing. Moreover, antigenic peptides showed a good overlap with previously identified T cell reactive areas. Therefore we suggest that the method might be used to predict T cell epitopes on allergenic proteins.

## Supplementary Materials

Supplementary materials can be found at www.mdpi.com/1422-0067/18/6/1225/s1.

## Abbreviations

ACN	Acetonitrile
AEP	Asparaginyl endopeptidase
APC	Antigen-presenting cell
CD	Cluster of differentiation
CLIP	Class II-associated invariant chain peptide
DC	Dendritic cell
DTT	Dithiothreitol
FA	Formic acid
FCSi	Fetal calf serum (heat inactivated)
GM-CSF	Granulocyte macrophage—colony stimulating factor
HLA	Human leucocyte antigen
LPS	Lipopolysaccharide
MHC	Major histocompatibility complex
MS	Mass spectrometry
PAGE	Polyacrylamide gel electrophoresis
RFU	Relative fluorescent unit
SDS	Sodium dodecyl sulfate
TFA	Trifluoroacetic acid

## References

[1] Delamarre L., Pack M., Chang H., Mellman I., Trombetta E.S. (2005). Differential lysosomal proteolysis in antigen-presenting cells determines antigen fate. Science.

[2] Jensen P.E. (2007). Recent advances in antigen processing and presentation. Nat. Immunol..

[3] Roche P.A., Furuta K. (2015). The ins and outs of MHC class II-mediated antigen processing and presentation. Nat. Rev. Immunol..

[4] Nitta T., Suzuki H. (2016). Thymic stromal cell subsets for T cell development. Cell. Mol. Life Sci..

[5] Valenta R., Ferreira F., Focke-Tejkl M., Linhart B., Niederberger V., Swoboda I., Vrtala S. (2010). From allergen genes to allergy vaccines. Annu. Rev. Immunol..

[6] Woodfolk J.A. (2007). T cell responses to allergens. J. Allergy Clin. Immunol..

[7] Egger M., Jurets A., Wallner M., Briza P., Ruzek S., Hainzl S., Pichler U., Kitzmuller C., Bohle B., Huber C.G. (2011). Assessing protein immunogenicity with a dendritic cell line-derived endolysosomal degradome. PLoS ONE.

[8] Mutschlechner S., Egger M., Briza P., Wallner M., Lackner P., Karle A., Vogt A.B., Fischer G.F., Bohle B., Ferreira F. (2010). Naturally processed T cell-activating peptides of the major birch pollen allergen. J. Allergy Clin. Immunol..

[9] Wallner M., Hauser M., Himly M., Zaborsky N., Mutschlechner S., Harrer A., Asam C., Pichler U., van Ree R., Briza P. (2011). Reshaping the Bet v 1 fold modulates TH polarization. J. Allergy Clin. Immunol..

[10] Rozman-Pungercar J., Kopitar-Jerala N., Bogyo M., Turk D., Vasiljeva O., Stefe I., Vandenabeele P., Bromme D., Puizdar V., Fonovic M. (2003). Inhibition of papain-like cysteine proteases and legumain by caspase-specific inhibitors: When reaction mechanism is more important than specificity. Cell Death Differ..

[11] Ghoneim H., Klinkert M.Q. (1995). Biochemical properties of purified cathepsin B from *Schistosoma mansoni*. Int. J. Parasitol..

[12] Huotari J., Helenius A. (2011). Endosome maturation. EMBO J..

[13] Jacquet A., Magi M., Petry H., Bollen A. (2002). High-level expression of recombinant house dust mite allergen Der p 1 in *Pichia pastoris*. Clin. Exp. Allergy.

[14] Derewenda U., Li J., Derewenda Z., Dauter Z., Mueller G.A., Rule G.S., Benjamin D.C. (2002). The crystal structure of a major dust mite allergen Der p 2, and its biological implications. J. Mol. Biol..

[15] De Halleux S., Stura E., VanderElst L., Carlier V., Jacquemin M., Saint-Remy J.M. (2006). Three-dimensional structure and IgE-binding properties of mature fully active Der p 1, a clinically relevant major allergen. J. Allergy Clin. Immunol..

[16] Chevigne A., Barumandzadeh R., Groslambert S., Cloes B., Dehareng D., Filee P., Marx J.C., Frere J.M., Matagne A., Jacquet A. (2007). Relationship between propeptide pH unfolding and inhibitory ability during ProDer p 1 activation mechanism. J. Mol. Biol..

[17] Chevigne A., Campizi V., Szpakowska M., Bourry D., Dumez M.E., Martins J.C., Matagne A., Galleni M., Jacquet A. (2017). The Lys-Asp-Tyr triad within the mite allergen Der p 1 propeptide is a critical structural element for the pH-dependent initiation of the protease maturation. Int. J. Mol. Sci..

[18] Takai T., Mizuuchi E., Kikuchi Y., Nagamune T., Okumura K., Ogawa H. (2006). Glycosylation of recombinant proforms of major house dust mite allergens Der p 1 and Der f 1 decelerates the speed of maturation. Int. Arch. Allergy Immunol..

[19] Bird P.I., Trapani J.A., Villadangos J.A. (2009). Endolysosomal proteases and their inhibitors in immunity. Nat. Rev. Immunol..

[20] Watts C., Matthews S.P., Mazzeo D., Manoury B., Moss C.X. (2005). Asparaginyl endopeptidase: Case history of a class II MHC compartment protease. Immunol. Rev..

[21] Manoury B., Mazzeo D., Fugger L., Viner N., Ponsford M., Streeter H., Mazza G., Wraith D.C., Watts C. (2002). Destructive processing by asparagine endopeptidase limits presentation of a dominant T cell epitope in MBP. Nat. Immunol..

[22] Vaithilingam A., Lai N.Y., Duong E., Boucau J., Xu Y., Shimada M., Gandhi M., Le Gall S. (2013). A simple methodology to assess endolysosomal protease activity involved in antigen processing in human primary cells. BMC Cell. Biol..

[23] Hofer H., Asam C., Hauser M., Nagl B., Laimer J., Himly M., Briza P., Ebner C., Lang R., Hawranek T. (2016). Tackling Bet v 1 and associated food allergies with a single hybrid protein. J. Allergy Clin. Immunol..

[24] Jahn-Schmid B., Wopfner N., Hubinger G., Asero R., Ebner C., Ferreira F., Bohle B. (2010). The T-cell response to Amb a 1 is characterized by 3 dominant epitopes and multiple MHC restriction elements. J. Allergy Clin. Immunol..

[25] Hoyne G., Bourne T., Kristensen N., Hetzel C., Lamb J. (1996). From epitopes to peptides to immunotherapy. Clin. Immunol. Immunopathol..

[26] Crack L.R., Chan H.W., McPherson T., Ogg G.S. (2012). Identification of an immunodominant region of the major house dust mite allergen Der p 2 presented by common human leucocyte antigen alleles. Clin. Exp. Dermatol..

[27] Neeno T., Krco C.J., Harders J., Baisch J., Cheng S., David C.S. (1996). HLA-DQ8 transgenic mice lacking endogenous class II molecules respond to house dust allergens: Identification of antigenic epitopes. J. Immunol..

[28] O’Brien R.M., Thomas W.R., Nicholson I., Lamb J.R., Tait B.D. (1995). An immunogenetic analysis of the T-cell recognition of the major house dust mite allergen Der p 2: Identification of high- and low-responder HLA-DQ alleles and localization of T-cell epitopes. Immunology.

[29] Verhoef A., Higgins J.A., Thorpe C.J., Marsh S.G., Hayball J.D., Lamb J.R., O’Hehir R.E. (1993). Clonal analysis of the atopic immune response to the group 2 allergen of *Dermatophagoides* spp.: Identification of HLA-DR and -DQ restricted T cell epitopes. Int. Immunol..

[30] O’Hehir R.E., Verhoef A., Panagiotopoulou E., Keswani S., Hayball J.D., Thomas W.R., Lamb J.R. (1993). Analysis of human T cell responses to the group II allergen of *Dermatophagoides* species: Localization of major antigenic sites. J. Allergy Clin. Immunol..

[31] Wu B., Elst L.V., Carlier V., Jacquemin M.G., Saint-Remy J.M. (2002). The *Dermatophagoides pteronyssinus* group 2 allergen contains a universally immunogenic T cell epitope. J. Immunol..

[32] Wambre E., Bonvalet M., Bodo V.B., Maillere B., Leclert G., Moussu H., von Hofe E., Louise A., Balazuc A.M., Ebo D. (2011). Distinct characteristics of seasonal (Bet v 1) vs. Perennial (Der p 1/Der p 2) allergen-specific CD4^+^ T cell responses. Clin. Exp. Allergy.

[33] Swoboda I., Jilek A., Ferreira F., Engel E., Hoffmann-Sommergruber K., Scheiner O., Kraft D., Breiteneder H., Pittenauer E., Schmid E. (1995). Isoforms of Bet v 1, the major birch pollen allergen, analyzed by liquid chromatography, mass spectrometry, and cDNA cloning. J. Biol. Chem..

[34] Chua K.Y., Huang C.H., Shen H.D., Thomas W.R. (1996). Analysis of sequence polymorphism of a major mite allergen, Der p 2. Clin. Exp. Allergy.

[35] Bond J.F., Garman R.D., Keating K.M., Briner T.J., Rafnar T., Klapper D.G., Rogers B.L. (1991). Multiple Amb a I allergens demonstrate specific reactivity with IgE and T cells from ragweed-allergic patients. J. Immunol..

[36] Thomas W.R. (2015). Hierarchy and molecular properties of house dust mite allergens. Allergol. Int..

[37] Kim K.J., Kanellopoulos-Langevin C., Merwin R.M., Sachs D.H., Asofsky R. (1979). Establishment and characterization of BALB/c lymphoma lines with B cell properties. J. Immunol..

[38] Raschke W.C., Baird S., Ralph P., Nakoinz I. (1978). Functional macrophage cell lines transformed by abelson leukemia virus. Cell.

[39] Jiang X., Shen C., Rey-Ladino J., Yu H., Brunham R.C. (2008). Characterization of murine dendritic cell line JAWS II and primary bone marrow-derived dendritic cells in *Chlamydia muridarum* antigen presentation and induction of protective immunity. Infect. Immun..

[40] Bradford M.M. (1976). A rapid and sensitive method for the quantitation of microgram quantities of protein utilizing the principle of protein-dye binding. Anal. Biochem..

