# Supplementary material for: Comparing Proteolytic Fingerprints of Antigen-Presenting Cells during Allergen Processing
Source: Int J Mol Sci. 2017 Jun 8;18(6):1225. doi: 10.3390/ijms18061225 (PMC5486048; doi:10.3390/ijms18061225)
Supplement: Supplementary file 1 [file ijms-18-01225-s001.pdf]

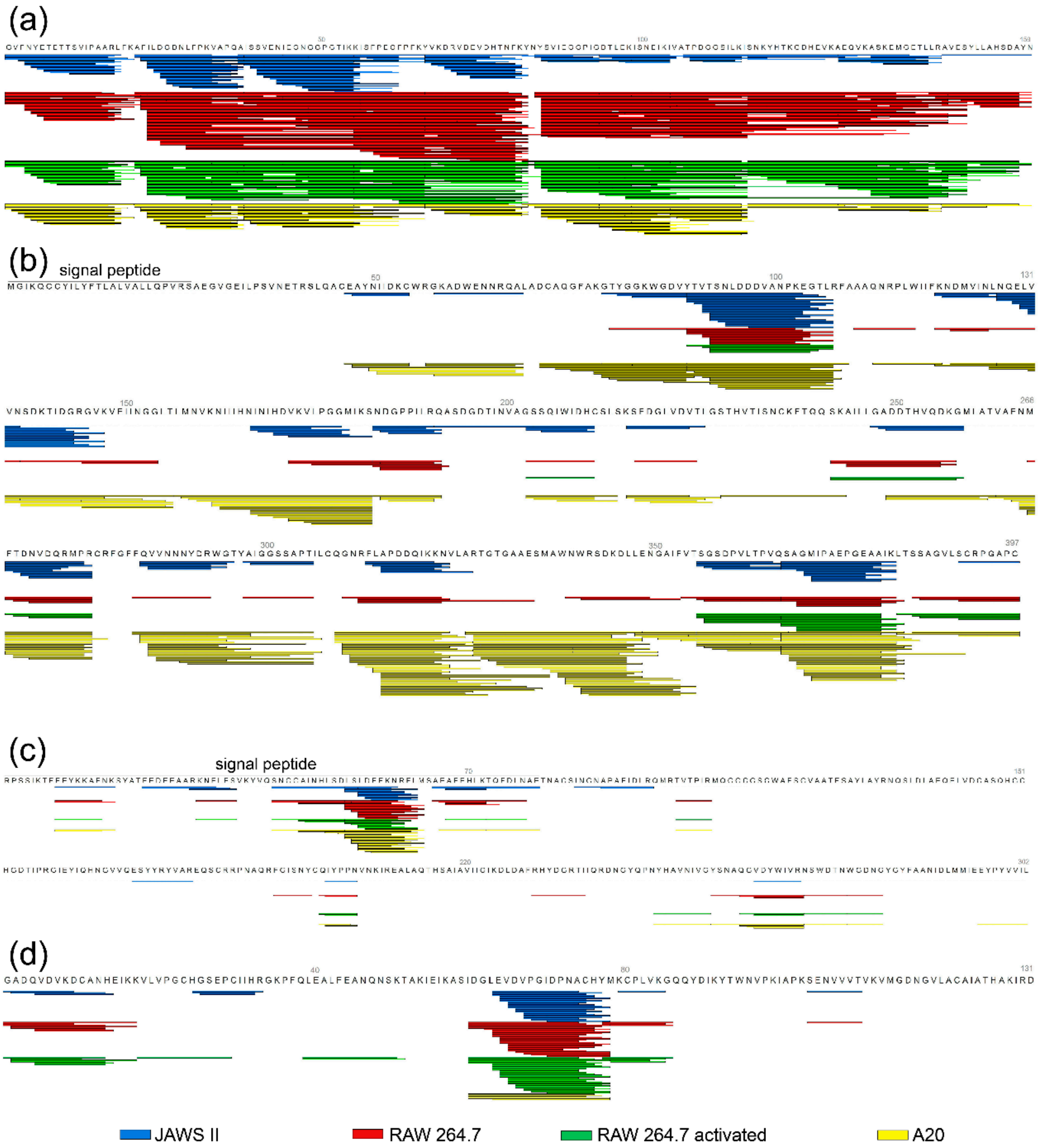

Figure S1. Endolysosomal degradations of purified allergens were analyzed by mass spectrometry (MS) after 24 h. Peptides identified by MS are represented as bars. The following protein sequences were used: Bet v 1.0101 (**a**); Amb a 1.0301 (**b**); proDer p 1.0102 (**c**); and Der p 2.0103 (**d**).
